# Bacteremia in Patients Undergoing Debridement, Antibiotics, and Implant Retention Leads to Increased Reinfections and Costs

**DOI:** 10.1016/j.artd.2022.05.014

**Published:** 2022-07-19

**Authors:** Samuel Rosas, Vishal Hegde, F. Johannes Plate, Douglas Dennis, Jason Jennings, Daniel N. Bracey

**Affiliations:** a Wake Forest Baptist Health, Department of Orthopaedic Surgery, Winston-Salem, NC USA; b Colorado Joint Replacement, Denver, CO, USA; c The Johns Hopkins University, Department of Orthopaedic Surgery, Baltimore, MD, USA; d University of Denver, Department of Mechanical and Materials Engineering, Denver, CO, USA; e University of Colorado School of Medicine, Department of Orthopaedic Surgery, Aurora, CO, USA; f University of Tennessee, Department of Biomedical Engineering, Knoxville, TN, USA; g University of North Carolina, Department of Orthopaedic Surgery, Chapel Hill, NC, USA

**Keywords:** Prosthetic joint infection, Arthroplasty, Infection, Total knee arthroplasty, Total hip arthroplasty

## Abstract

**Background:**

Debridement, antibiotics, and implant retention (DAIR) is a common treatment for acute prosthetic joint infection (PJI). The effects of concurrent bacteremia at the time of DAIR are poorly understood. This study sought to determine whether patients with bacteremia at the time of DAIR have higher reinfection rates.

**Material and methods:**

A retrospective review of a national database was performed. Patients treated with DAIR (hip or knee arthroplasty) after a diagnosis of PJI were identified. DAIR patients who also had a diagnosis of bacteremia were matched to patients without bacteremia by comorbidities and Charlson Comorbidity Index score. The primary outcome was reinfection or continued infection at 90 days and 6, 12, and 24 months after DAIR. Ninety-day Medicare charges were compared between groups. Survival probabilities were used for survival comparisons.

**Results:**

A total of 9945 patients underwent DAIR after a diagnosis of PJI. Seven hundred seven patients underwent DAIR with an associated diagnosis of bacteremia. Three hundred thirty-four DAIR patients with bacteremia were successfully matched to patients without bacteremia by age, gender, and comorbidities. DAIR survivorship was significantly worse in those with bacteremia at 90 days (51.5% vs 65.9%) and 6 (43.1% vs 60.5%), 12 (36.5% vs 56.0%), and 24 months (32.6% vs 53.3%) after DAIR. The 90-day costs of DAIR were significantly greater in PJI patients with bacteremia (mean: $14,722 standard deviation: $4086 vs mean: $8,052, standard deviation: $4,153, *P* < .01).

**Conclusions:**

Patients undergoing DAIR with bacteremia are at an increased risk of reinfection or continued infection. Ninety-day costs are significantly increased (over 50%) in patients with bacteremia vs those without bacteremia.

## Introduction

With the rising number of hip and knee arthroplasties, a concomitant increase in the number of complications including prosthetic joint infections (PJIs) is expected. Various treatment modalities for PJI are available based on patient comorbidities, time since arthroplasty, patient-specific risk factors for further complications, and overall health status. Current treatment options include single-stage revision, 2-stage revision, chronic antibiotic suppression, or debridement with irrigation, antibiotics, and implant retention (DAIR). DAIR is commonly utilized in patients medically unfit to undergo a 2-stage revision and for those with acute-onset PJI.

Retrospective Medicare data presented by Boyle et al. showed increasing utilization of DAIR in treatment of PJIs in the US in total knee arthroplasty (TKA) patients with a high number of comorbidities and of older age [1]. The reported success rate with DAIR has been highly variable, ranging anywhere from 16% [2] to 77% [3]. These studies highlight our inability to predict which PJI patients can be successfully treated with DAIR [4]. One patient variable given closer attention in recent years is the presence of bacteremia at the time of DAIR. Two previous cohort studies of 22 and 43 patients showed that positive blood cultures decrease treatment success of PJIs [5,6]. To date, no study has utilized Medicare database queries to determine if bacteremia decreases success of DAIR for acute PJIs, and no study has investigated the potentially increased PJI costs in this setting. The current study cohort of 334 DAIR patients with concurrent bacteremia is the largest reported in any literature.

The purpose of this study was to evaluate whether patients undergoing DAIR for PJI after total hip arthroplasty (THA) or TKA are at an increased risk of treatment failure compared to those without bacteremia. We hypothesized that bacteremia, defined by positive blood cultures at the time of DAIR for THA or TKA PJI, would adversely affect outcomes of DAIR leading to decreased survivorship in that patient cohort compared to DAIR patients without bacteremia. Similarly, we hypothesized that the presence of bacteremia would significantly increase costs associated with PJI treatment.

## Material and methods

A retrospective case-control study was conducted utilizing a commercially accessible server to query the Medicare Dataset of the Standard Analytical Files. Institutional review board approval from our institution was obtained prior to this study. This dataset contains the entire patient population of Medicare patients during the duration of the patients’ enrollment in Medicare. Briefly, the PearDiver Server (Boulder, CO) is a commercially available server that houses patient records in a Health Insurance Portability and Accountability (HIPAA)–compliant fashion. The server houses data from private payers and Medicare and allows for longitudinal evaluation of patient cohorts. Patients were identified through International Classification of Disease 9th Revision (ICD-9) and ICD-9 procedure codes (Appendix 1). The current study utilized the Medicare dataset housed within the server given that most arthroplasties occurring in the United States are performed in patients over the age of 65. The dataset contains over 55 million patient records from 2005 to 2014 which represents 100% of the Medicare sample.

### Study cohort identification

**Figure 1:**
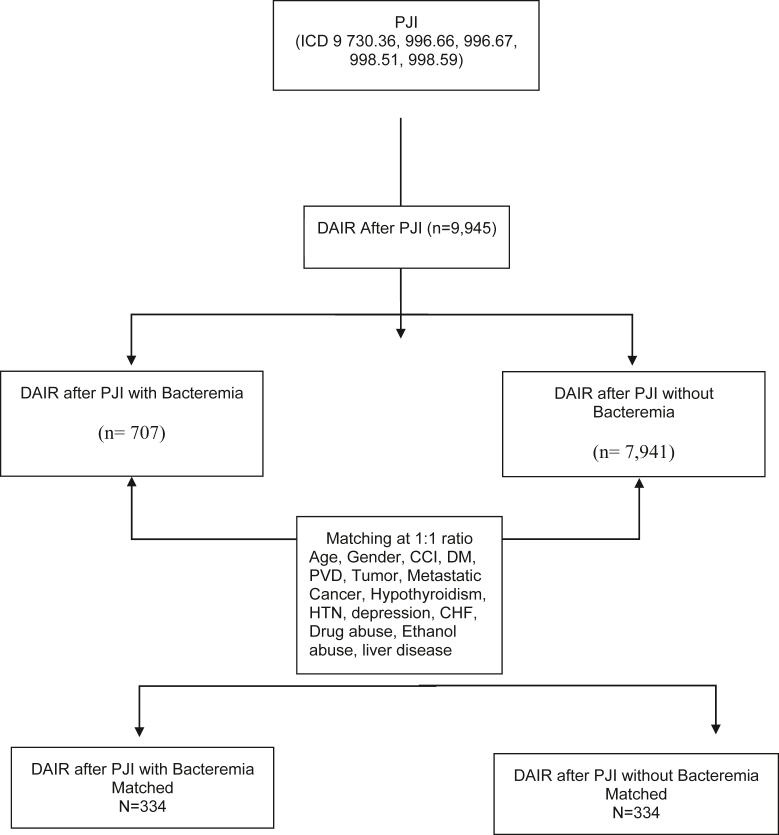
CONSORT flow diagram demonstrating study characteristics.

Patients with a diagnosis of PJIs were identified in the database based on ICD codes. From this cohort, patients who underwent DAIR for THA (revision with femoral head and acetabular liner exchange) or TKA (revision with tibial insert exchange) were extracted by ICD-9 procedure codes (Appendix 1). Patients with bacteremia at the time of DAIR for PJIs were then identified. These patients were matched to a cohort of patients who underwent DAIR for the same indication without bacteremia based on age, gender, comorbidities, and Charlson Comorbidity Index (CCI) score. Patients were matched to control for comorbidities believed to increase risk of infection or limit a patient’s ability to clear infection with surgical debridement. For example, it is well known that alcohol abuse has been linked to worse outcomes after TJA [7] or that patients with HIV have an increased risk of deep vein thrombosis [8]. We attempted to match patients on these comorbidities to decrease effects of confounding comorbidities. We recognized this would limit our ultimate sample size but felt it was necessary for appropriate cohort comparison in a study already limited by claims-based data. Other comorbidities that have been previously correlated with PJIs were thus also included [[9], [10], [11], [12], [13]]. Figure 1 demonstrates the study design as suggested by the CONSORT guidelines. Costs were evaluated based on Medicare reimbursements. This is a previously used method of describing costs that allows for external description of expenditure by Medicare [14]. The 90-day costs were used based on current bundled payment initiatives. Survival was assessed as a new diagnosis of PJIs after the DAIR was performed. Endpoint assessment was performed at 90 days and 6, 12, and 24 months. This study was designed to comply with the recently published guidelines for database studies published by the leadership of the Journal of Arthroplasty [15].

### Statistical evaluation

The statistical package of R study available within the PearlDiver server was used to conduct multivariate and univariate analysis. Parametric and nonparametric testing on continuous data was performed with SPSS, version 20 (IBM Corp, Armonk, NY) and by way of students t-tests and Mann-Whitney tests. Chi-Square testing was used to compare percentage of individual comorbidities within the matched cohorts. A multivariate regression was conducted to determine whether bacteremia was associated with reinfection when accounting for age, gender, and CCI. Finally, Kaplan-Meier survival curves were used to assess survival of surgery after DAIR. Survival was defined as the time free of reinfection from the date of DAIR until a new PJI diagnosis was identified by a new ICD-9 code.

## Results

### Study population

Within the Medicare records, 73,435 patients who underwent modular component exchange of a THA or TKA were identified between the years of 2005 and 2014. During that same time period, 1,519,749 patients had a diagnosis of PJIs and 9945 patients underwent DAIR after a diagnosis of PJIs. Ultimately, 707 patients (7.1%) were diagnosed with bacteremia at the time of DAIR. Of these 707 patients, 334 were successfully matched by age, gender, and comorbidities to patients undergoing DAIR without concurrent bacteremia.

### Study cohort characteristics

Each cohort was comprised of 43% females. The majority of patients were aged 65 to 69 years (28%), and those aged 64 years and younger comprised 25%. Table 1 demonstrates the characteristics of the patients included in the final cohort. Of note, gender, age, and region where the procedure took place were all similar within the 2 compared cohorts (*P* > .05 for all). Similarly, when comparing the distribution of 21 comorbidities within the 2 groups, we found no significant differences between both patient cohorts, demonstrating a successful matching process (Table 2, *P* > .05 for all).

**Table 1:** Study demographic characteristics and group comparisons.

Demographics	Bacteremia	No bacteremia	Chi-square
Gender			1
Females	191	191	
Males	143	143	
Age			0.9988
64 and under	85	82	
65-69	95	95	
70-74	61	62	
75-79	59	58	
80-84	27	30	
85 and over	7	7	
Region			0.07463
Midwest	109	94	
Northeast	71	53	
South	104	126	
West	50	61

**Table 2:** Comorbidity prevalence comparisons among study groups.

Prevalence of comorbidities	Bacteremia	No bacteremia	Chi-square
AIDS	0.30%	0.30%	1
Blood loss anemia	24.9%	18.6%	0.061
CHF	43.4%	43.4%	1
Depression	60.2%	60.2%	1
DM	53.0%	53.0%	1
Drug abuse	10.8%	10.8%	1
Ethanol abuse	5.7%	5.7%	1
Hypertension	97.0%	97.0%	1
Hypothyroidism	32.3%	32.3%	1
Liver disease	7.5%	7.5%	1
Lymphoma	3.9%	2.1%	0.256
Metastatic cancer	0.0%	0.0%	1
Neurological disorders	15.6%	14.7%	1
Obesity	0.0%	0.0%	1
Paralysis	5.4%	7.2%	0.425
Psychotic disorders	26.6%	21.3%	0.123
PUD	15.0%	15.6%	1
Pulmonary disease	55.4%	59.0%	1
PVD	41.6%	41.6%	1
Rheumatologic diseases	29.9%	26.3%	0.343
Tumors	22.2%	22.2%	1
Mean CCI	5.34	5.34	1
SD CCI	2.16	2.16	

### Outcome comparison

**Figure 2:**
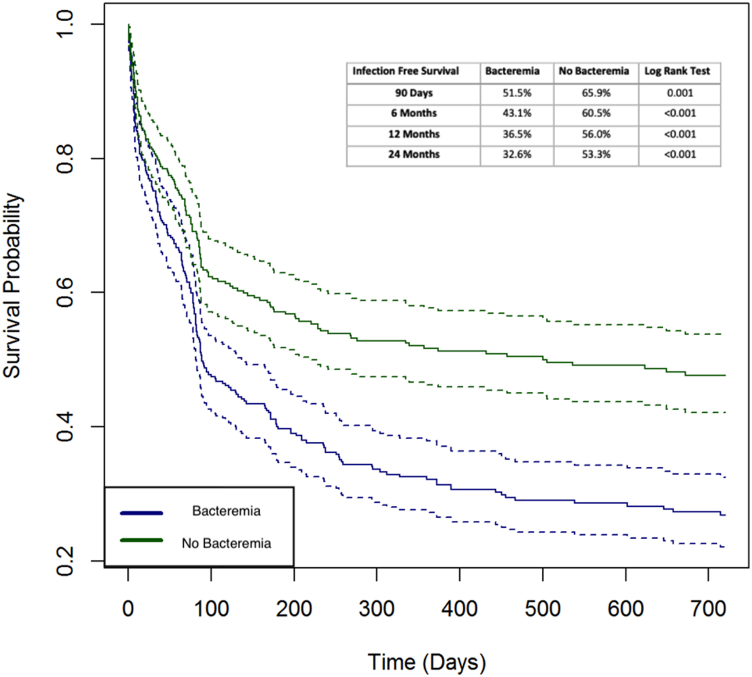
Kaplan-Meier survival curves. Patients with and without bacteremia at the time with 95% confidence intervals (dashed lines).

The mean inpatient length of stay in patients undergoing DAIR with bacteremia (6.47 days, standard deviation [SD]: 2.3) was significantly greater than that in patients without bacteremia (3.83 days, SD: 1.06, *P* = .004). The multivariate regression analysis demonstrated that age under 65 years and age over 84 years were associated with decreased risk of reinfection following DAIR for PJIs, while increasing CCI, male gender, and bacteremia at the time of DAIR were all significant predictors of reinfection. Table 3 demonstrates the adjusted odds ratios for reinfection after DAIR with the respective 95% confidence intervals. Most notably, bacteremia at the time of DAIR had the highest odds ratio (OR) at 24 (95% CI: 18.37 – 31.45). Similarly, comparative survivorship curves between the 2 patient cohorts (Fig. 2) showed significantly worse survivorship in bacteremic patients at 90 days (51.5% vs 65.9%, *P* = .001) and 6 (43.1% vs 60.5%, *P* < .001), 12 (36.5% vs 56.0%, *P* < .001), and 24 months (32.6% vs 53.3%, *P* < .001) (Table 4).

**Table 3:** Multivariate comparison of risk factors to failure.

Variable	aOR	2.50%	97.50%	*P* value	Significance
(Intercept)	0.44	0.31	0.64	.000	∗
Age, years					
65-69	0.64	0.44	0.92	.015	∗
70-74	0.72	0.49	1.07	.104	
75-79	0.69	0.47	1.03	.068	
80-84	0.45	0.30	0.68	.000	∗
>84	0.39	0.25	0.62	.229	
CCI	1.10	1.05	1.16	.000	∗
Male	1.39	1.11	1.73	.004	∗
Bacteremia	24.03	18.37	31.45	.000	∗

**Table 4:** Infection free survival rates.

Infection-free survival	Bacteremia	No bacteremia	Log-rank test
90 d	51.5%	65.9%	0.001
6 mo	43.1%	60.5%	<0.001
12 mo	36.5%	56.0%	<0.001
24 mo	32.6%	53.3%	<0.001

Reimbursement comparison demonstrated that mean reimbursements for those who were bacteremic at the time of DAIR were significantly greater (mean: $14,722, SD: $4086 vs mean: $8,052, SD: $4,153, *P* = .001), representing an increase of 183% with an annual variation from 121% to 406%.

## Discussion

The current study sought to identify whether bacteremia at the time of DAIR for treatment of PJIs was associated with increased reinfection rates and costs within a cohort of Medicare patients treated in the United States. The failure rate of DAIR in patients with bacteremia was 49.5% at 90 days compared to 35.1% in those without bacteremia. The failure rate in the bacteremia cohort continued increasing to as high as 67.4% at 2 years vs 47.7% in the matched cohort. The current study adjusted for 21 confounding variables that have previously been suggested to increase the risk of failure following DAIR and ultimately found that concurrent bacteremia is a single independent risk factor for failure after DAIR (odds ratio: 24.03).

Treatment of PJIs with DAIR has previously been addressed, with most literature focusing on how treatment success rates correlate with virulence of the isolated pathogen or host comorbidities [16,17]. Only a limited subset of literature has previously considered how bacteremia at the time of DAIR may affect success rates of PJI treatment. Klement et al. retrospectively reviewed the records of 320 patients treated for THA and TKA PJI at 2 academic institutions and found that blood cultures were obtained in 57% of patients [6]. In the 43 patients with positive blood cultures, blood and synovial culture data matched in 82% of cases. Logistic regression analysis showed that decreasing treatment successes was associated with increased comorbidity index, 2-stage treatment, and positive blood culture at the time of treatment. Treatment success was only 65.1% in those with positive blood cultures (n = 43) compared to 85% in blood culture–negative patients (*P* = .013). Positive blood cultures were associated with what the authors considered a greater disease burden indicated by higher synovial white blood cell counts, higher serum C-reactive protein levels, and higher mortality rates than the nonbacteremic cohort.

A similar study by Kuo et al. [5] used a similar retrospective design as Klement et al. [6] but focused their hypothesis on PJI patients treated specifically with DAIR. Preoperative blood cultures were obtained in 49 acute PJI patients treated with DAIR, and 22 of these patients (45%) had positive blood cultures. Patients with positive blood cultures again had higher comorbidity indices and elevated WBC counts compared the PJI patients with negative blood cultures, similar to the findings by Klement et al. DAIR treatment success 1 year postoperatively was significantly lower in bacteremic patients (36.3%) than in patients with negative blood cultures (66.7%, *P* = .047). Their analysis found that positive blood cultures, polymicrobial infections, and elevated comorbidity indices were all significant predictors of failed treatment, but after stepwise multivariate logistic regression analysis, only positive blood cultures were a significant predictor of failed treatment.

The current study presents the largest cohort of PJI patients treated with DAIR in the setting of concurrent bacteremia and is the first investigation to pool Medicare data for this study population. The failure rates described in our study are higher than those previously reported. Our study reported 2-year outcomes, included only patients treated with DAIR and excluded PJI patients treated with 2-stage procedures which are traditionally more successful in eradicating infections than DAIR. Additionally, the data presented by Kuo et al. focused on acute hematogenous PJI diagnosed within 3 months of the index procedure, while our dataset included all patients treated with DAIR, which could include either acute or chronic PJI. Consistent with the conclusions of Klement et al and Kuo et al., we found that DAIR in the setting of bacteremia results in significantly worse survivorship. Our 2-year survivorship rates are the longest reported to date. The limited success of DAIR in patients with bacteremia may encourage providers to more routinely obtain blood cultures when evaluating PJI patients to better risk stratify which patients are more appropriate for DAIR vs 2-stage exchange. While we previously believed that treatment success was largely dependent on virulence of the isolated organism, more recent data could suggest that the disease burden in the setting of bacteremia is a more relevant predictor of treatment success. Further study is required to understand the timing of bacteremia and effects on eradication of the infection. Patients may benefit from clearance of bacteremia prior to PJI debridement and should only undergo definitive DAIR once they have negative blood cultures. Alternatively, bacteremic patients may be indicated for 2-stage revision regardless of blood cultures being positive before or after DAIR.

The current study found increased costs in the 90-day episode of care for patients with bacteremia. This finding reveals the expected increase in resource utilization required to care for these patients. Multiple consulting services, possibly higher-level of care, long-term intravenous antibiotics, additional surgeries, additional implants, and greater use of resources in both the inpatient and outpatient care settings, all significantly increase healthcare expenditure. No previous study has assessed differences in reimbursement between PJI patients undergoing DAIR with or without bacteremia. The findings of the current study should alert policymakers and practice leaders about this resource-intense cohort of patients. Furthermore, the cost evaluation in this study can help future studies establish cost-effectiveness of DAIR vs single-stage exchange vs 2-stage exchange in certain patients.

### Limitations

The results of this retrospective study should be interpreted recognizing the inherent limitations of a large database analysis. The cohort of patients with bacteremia was identified through claims-based diagnosis and procedural codes that are subject to error in coding, overcoding and/or under-coding. Without access to medical records and operative notes, we are limited in what we know about the severity of infection, extent of debridement, and clinical decision-making. For example, without access to the blood culture data, we are unable to determine if bacteremia was always diagnosed with positive blood cultures. Additionally, we are unable to know when blood cultures were taken and what percentage of patients undergoing DAIR had blood cultures. All patients undergoing DAIR were included in our cohort, and we were unable to determine if this was performed for acute PJI, acute hematogenous seeded PJI, or possibly chronic PJI. Acuity of infection would likely confound the efficacy of DAIR in the setting of bacteremia. Furthermore, the retrospective nature of this study and the relatively limited sample size are also factors to consider when interpreting results of this study. Our ultimate sample size was significantly reduced after matching, but we felt appropriate matching was necessary in a study already limited by claims-based data. We did not have access to the culture data to accurately assess effects of different organisms on treatment success. Additionally, there were no standardized criteria for obtaining blood cultures which is a limitation inherent to all retrospective studies previously published on this topic. Reimbursement data may also fail to capture a large portion of the indirect healthcare-related costs associated with care of these patients attributed to the long recovery process and inability to work. Also, the nature of Medicare reimbursement analysis decreases the ability to compare costs between payers. Other medical and social determinants of outcomes such as time to infection, microorganism involved, and host factors such as immune status were not evaluated and could potentially alter our findings. This is the largest matched study cohort to date, however, and findings are consistent with previous reports.

## Conclusions

Patients with PJI with concurrent bacteremia at the time of DAIR have worse survivorship and incur increased costs during the episode of care compared to a comorbidity matched cohort of PJI patients undergoing DAIR without bacteremia. Additional study is needed to determine if bacteremia patients are indicated for 2-stage exchange or if they would still be appropriate for DAIR after their bacteremia has resolved.

## Appendix A Supplementary data

Conflict of Interest Statement for Plate

Conflict of Interest Statement for Dennis

Conflict of Interest Statement for Jennings

Conflict of Interest Statement for Rosas

Conflict of Interest Statement for Bracey

Conflict of Interest Statement for Hegde

## Appendix 1

List of ICD-9 codes used for this study:

996.66 Infection due to internal joint prosthesis
996.67 Infection due to other internal orthopaedic device, implant and graft
998.51 Infected postoperative seroma
998.59 Other postoperative infection
730.36 Periostitis without mention of osteomyelitis, lower leg
008.4 Other specified bacteria
790.7 Bacteremia
796.4 Other abnormal clinical findings
795.39 Nonspecific positive culture findings
List of ICD-9 Procedure codes used for this study:
0073 Revision of total hip replacement acetabular liner and/or femoral head only 0084 Revision of total knee replacement tibial insert (liner) only
