# Supplementary material for: Bacteremia in Patients Undergoing Debridement, Antibiotics, and Implant Retention Leads to Increased Reinfections and Costs
Source: Arthroplast Today. 2022 Jul 19;16:259–263.e1. doi: 10.1016/j.artd.2022.05.014 (PMC9458898; doi:10.1016/j.artd.2022.05.014)
Supplement: Conflict of Interest Statement for Plate [file mmc1.pdf]

INDIVIDUAL CONFLICT OF INTEREST STATEMENT

American Association of Hip and Knee Surgeons

(Adopted from the American Academy of Orthopaedic Surgeons disclosure statement)

The following form **must be filled out completely and submitted by each author (example, 6 authors, 6 forms). All items require a response. If there is no relevant disclosure for a given item, enter "*None*."**

Manuscript Title:

---

1. Royalties from a company or supplier (The following conflicts were disclosed) - NONE

2. Speakers bureau/paid presentations for a company or supplier (The following conflicts were disclosed) - NONE

3A. Paid employee for a company or supplier (The following conflicts were disclosed) - NONE

3B. Paid consultant for a company or supplier (The following conflicts were disclosed) – Total Joint Orthopedics, Smith & Nephew

3C. Unpaid consultants for a company or supplier (The following conflicts were disclosed)

4. Stock or stock options in a company or supplier (The following conflicts were disclosed) – Eventum Orthopaedics

5. Research support from a company or supplier as a Principal Investigator (The following conflicts were disclosed) – Biocomposites Inc., Aerobiotix Inc.

6. Other financial or material support from a company or supplier (The following conflicts were disclosed) - NONE

7. Royalties, financial or material support from publishers (The following conflicts were disclosed) - VisualDX

8. Medical/Orthopaedic publications editorial/governing board (The following conflicts were disclosed) – Journal of Arthroplasty

9. Board member/committee appointments for a society (The following conflicts were disclosed) - NONE

Each author must sign AND print or type his/her name, date and submit a separate form

In addition, one BLINDED Conflict of Interest form (no author names used) should be submitted per manuscript with all author disclosures.

Johannes F. Plate, MD, PhD	J. Plate	6/5/21
Author Name (Print or Type)	Author Signature	Date
