# Supplementary material for: Bacteremia in Patients Undergoing Debridement, Antibiotics, and Implant Retention Leads to Increased Reinfections and Costs
Source: Arthroplast Today. 2022 Jul 19;16:259–263.e1. doi: 10.1016/j.artd.2022.05.014 (PMC9458898; doi:10.1016/j.artd.2022.05.014)
Supplement: Conflict of Interest Statement for Jennings [file mmc3.docx]

# INDIVIDUAL CONFLICT OF INTEREST STATEMENT

***American Association of Hip and Knee Surgeons***

(Adopted from the American Academy of Orthopaedic Surgeons disclosure statement)

The following form **must be filled out completely and submitted by each author (example, 6 authors, 6 forms).**

**All items require a response. If there is no relevant disclosure for a given item, enter "*None*.”**

**Manuscript Title:**

1. Royalties from a company or supplier (The following conflicts were disclosed)

None

2. Speakers bureau/paid presentations for a company or supplier (The following conflicts were disclosed)

None

3A. Paid employee for a company or supplier (The following conflicts were disclosed)

None

3B. Paid consultant for a company or supplier (The following conflicts were disclosed)

Total Joint Orthopedics; Xenex

3C. Unpaid consultants for a company or supplier (The following conflicts were disclosed)

None

4. Stock or stock options in a company or supplier (The following conflicts were disclosed)

Xenex

5. Research support from a company or supplier as a Principal Investigator (The following conflicts were disclosed)

DePuy, A Johnson & Johnson Company; Corin U.S.A**;** Porter Adventist Hospital

6. Other financial or material support from a company or supplier (The following conflicts were disclosed)

None

7. Royalties, financial or material support from publishers (The following conflicts were disclosed)

None

8. Medical/Orthopaedic publications editorial/governing board (The following conflicts were disclosed)

None

9. Board member/committee appointments for a society (The following conflicts were disclosed)

None

**Each author must sign AND print or type his/her name, date and submit a separate form**

In addition, one BLINDED Conflict of Interest form (no author names used) should be submitted per manuscript with all author disclosures.

Jason Jennings, MD Jason Jennings 3/30/2021

Author Name (Print or Type) Author Signature Date
